# Impact of the COVID-19 pandemic on the food rations of refugees in Rwanda

**DOI:** 10.1186/s12939-021-01450-1

**Published:** 2021-04-26

**Authors:** Emery Manirambona, Theogene Uwizeyimana, Emmanuel Uwiringiyimana, Henna Reddy

**Affiliations:** 1 College of Medicine and Health Sciences, University of Rwanda, Kigali, Rwanda; 2 Department of Public Health, Mount Kenya University Rwanda, Kigali, Rwanda; 3 Medical Sciences Division, University of Oxford, Oxford, UK

**Keywords:** COVID-19, Rwanda, Great Lakes, Refugees, Nutrition, Humanitarian assistance, Health equity

## Abstract

The coronavirus disease (COVID-19) has significantly impacted the global economy, by forcing people to stay indoors and creating a ‘new normal’ of living. Rwanda has made notable efforts to fight the pandemic. However, the impacts of the COVID-19 pandemic on the country’s economy are numerous and the refugees residing in Rwanda are not spared these effects. As of December 2020, 164,000 people were granted refugee status in Rwanda according to the United Nations High Commissioner for Refugees (UNHCR). The majority were from neighbouring countries in the Great Lakes regions, including DRC (Democratic Republic of Congo) and Burundi. The impact the COVID-19 pandemic on the global economy has led to a decline in donations to the United Nations World Food Programme (WFP), which in turn has significantly reduced the food rations of refugees. Such paucity will no doubt cause unprecedented impacts on the people residing in refugee camps, who completely depend on humanitarian aid to meet their basic food requirements. This lack of access to adequate and affordable food will expose refugees to extreme hunger and starvation, putting their lives in danger by triggering forced returns, infections, social conflicts and thus higher morbidity and mortality.

Furthermore, such stressful environments would no doubt put the mental health of these already vulnerable communities at risk. It is unsurprising that refugees are more likely to experience poor mental health compared to local population, including higher rates of depression and anxiety disorders including Post-Traumatic Stress Disorder (PTSD). This is an issue as they are also less likely to receive support than the general population. Refugees in Rwanda are under the responsibility of UNHCR and WFP, who should ensure adequate food assistance is provided to refugees and therefore ameliorate the risks to health that result from food shortages, safeguarding these vulnerable communities.

## Background

The coronavirus disease (COVID-19) has significantly impacted the global economy, by forcing people to stay indoors and creating a ‘new normal’ of living. The impacts of COVID-19 are far-reaching and food security is not an exception. In March 2020, the World Health Organization (WHO) declared the COVID-19 a global pandemic [1], and many countries were ill-prepared to deal with this novel virus. Rwanda, a low-income country located in East Africa, established a national committee composed of members of different ministries, adopting a whole government approach to fight this global enemy. This was done right before the first case of COVID-19 was documented in Rwanda on March 14th 2020, and immediate preventive measures were devised including closure of schools, churches and any other mass gatherings [2]. As the number of cases continued to surge, the government imposed a countrywide lockdown as of 22 March 2020 [3], and multiple stringent precautionary measures were enforced. These included but are not limited to frequent hand washing, avoiding hand shaking, staying at home, avoiding unnecessary travels and avoiding mass gathering. The government established a social protective plan that aimed at providing support to the most vulnerable groups of Rwandan society, whereby a door-to-door delivery of foods and materials for other basic needs were freely distributed [4].

On May 4th 2020, the government relaxed the lockdown measures allowing people to resume businesses and movements while still respecting other mandated measures including practicing social distancing, wearing a mask, temperature monitoring services and hand hygiene stations at the entrances of all resumed services, as well as curfew hours from 8:00 pm to 4:00 am [5]. However, lockdown measures were implemented again in January 2021, following the rise in cumulative cases from 2.6 to 7.7%.

Although Rwanda has the most trusted healthcare system by its citizens globally according to a Wellcome Trust report [6], it has still struggled under the weight of the COVID-19 pandemic, with 17,929 cumulative cases and 245 deaths registered as of 20/02/2021.

Of particular impact is the decline in donations to the World Food Program (WFP) resulting from the detrimental effect the COVID-19 pandemic has had on the global economy. This has in turn significantly reduced food rations provided to refugees in Rwanda, and the WFP has announced a policy that will cut 60% of food rations to refugees in Rwanda. In this paper, we aim to discuss the numerous adverse effects such ration reductions could have on refugees in Rwanda, with the hope this will lead to reconsideration of this detrimental ration reduction and promote discussion for a sustainable solution to this crisis in health equity.

## Refugee situation in Rwanda

After the 1994 Genocide Against the Tutsi in Rwanda, that took the life of over 1 million Tutsis along with thousands of displaced people, Rwanda has made notable progress and established strong leadership that has stabilised the country [7]. People who faced political or ethnic crisis in neighbouring countries came to seek protection and asylum in Rwanda and, thus, it has been hosting refugees for over 20 years. Further, at the end of 2020, the United Nations High Commissioner for Refugees (UNHCR) counted 164 thousand people who were granted refugee status in Rwanda, whose majority were from neighbouring countries in the Great Lakes regions including DRC (Democratic Republic of Congo) and Burundi. Interestingly, Rwanda does not only host refugees from the Great Lakes region but also from the entire African continent. A total number of 385 persons, both asylum seekers and refugees in Libya, were evacuated to Rwanda. Needless to say, Rwanda alone cannot provide all the necessary protection and resources refugees require [8]. An inclusive coordination that comprises the UNHCR, World Food Program (WFP) and the Rwandan government, along with other partners and agencies, play an indispensable role to ensure that refugees are protected, and their basic needs are met, in particular guaranteeing food, health and education are provided [9].

However, the COVID-19 pandemic, a major public health concern globally, has seriously affected refugees in Rwanda. On the 8th of February 2021, a communique [9] surprised refugees in the Mahama camp occupied by Burundians [10], who fled their country following the political crisis due to the controversial third term of the Burundian president Peter Nkurunziza in 2015 [11]. Signed by WFP’s agency in Rwanda in partnership with UNHCR and the ministry in charge of refugees, amid the strict restrictions within the COVID-19 pandemic and lockdown, the communique stated a 60% cut of food aid to refugees in Rwanda followed by a scheme for unconditional cash transfers [9, 12, 13]. The refugee community in Rwanda called this “bad news”, given the fact that even the cash they used to buy food was far from enough to satisfy their needs, and this created sentiments of fear among them [14, 15].

In fact, each refugee in Rwanda receives a monthly sum of 7600 Rwandans Francs, equivalent to 7.72 United States Dollars, to buy food at local markets. However, this has never been enough to satisfy their individual minimum food needs, since this sum itself was a result of a recent cut of 25% on refugees’ rations in 2018 [12].

If in March 2021 WFP implements the new policy to cut the refugees’ food rations as announced, there would be multitudinous unprecedented impacts, particularly to the people residing in refugee camps who depend heavily on humanitarian aid to meet their basic food needs. Lack of food will have detrimental consequences to the life of these people, who have already suffered mental and physical wounds from conflicts in their countries of origin [16]. Poor nutrition will influence the mental health of the refugees and cause psychological distress. Further, food insecurity has an established correlation with mental illness, mainly anxiety and depression [17].

People sought asylum in Rwanda because they had a founded fear of living in or returning to their countries of birth. However, there is a likelihood that some might be forced to return to the countries they fled because they cannot afford basic necessities such as food, either for themselves or for their families. Here, a refugee from Burundi reacted: “Hmmm! No doubt I will have to go back because I prefer to die at home. I must feed my 4 children, my husband is in prison in Burundi, the camp is closed, and I cannot even go out to do daily chores like before the COVID-19 pandemic. Something like this only reminds me of the ordeal I had to endure to get here.” [14]. Compounded by disruption of the food supply chain in Rwanda caused by the COVID-19 financial crisis, a lack of access to adequate and affordable food will expose refugees to extreme hunger, putting their lives in danger by triggering forced returns.

Further, this could block other people who are seeking asylum in Rwanda, since the instability of the Great Lakes region and other countries in Africa is ongoing, and will undoubtedly result in suffering and deaths which could be avoided.

Equally important to consider is high quality nutrition, as a balanced diet and food sufficient in calories is indispensable in maintaining health and preventing disease that results from poor nutrition. Relevant to this point, the severe food insufficiency in refugee camps will have numerous effects, including but not limited to undernourishment, vitamin deficiency, high rates of infections [18] and even possibly social conflicts. It is worth noting that malnutrition is a public health issue that concerns all ages and people, which can worsen COVID-19 infection and has been proved to be a risk factor for death [18].

WFP plays a noteworthy role in achieving Sustainable Development Goal (SDG) 2: zero hunger. Therefore, it has a consequential responsibility in the COVID-19 response to provide food assistance and mitigate potential risks that would result from food shortages, particularly in the poorest and most vulnerable communities like refugees, who entirely depend on humanitarian assistance. Since WFP has pledged to “end hunger, achieve food security and improve nutrition”, we recommend that WFP takes immediate action and refrains from implementing the policy that proposes to cut 60% of food rations to refugees in Rwanda. Additionally, it should ensure refugees have access to adequate and affordable food, as the current scarcity is itself an emergency. Otherwise this UN Agency, would have failed in its mission to provide food security in such vulnerable refugee communities, which would also violate their human rights and make refugees more susceptible to communicable diseases [19], notably COVID-19.

## Conclusion

The global threat of COVID-19 has had tremendous consequences in all areas of life. Rwanda has taken exemplary action against the COVID-19 outbreak, saving countless lives, but the effects of the pandemic have inevitably been detrimental to the country’s economy and significantly impacted the food supply chain. Rwanda hosts thousands of refugees from the Great Lakes region. A strong partnership between the Rwandan government and WFP, UNHCR and other UN agencies along with other stakeholders has strengthened the functionality of the refugee system and improved the refugees’ lives. However, the new policy to reduce refugees’ food rations by 60%, which follows a recent food cut of 25%, would have immeasurable consequences on this vulnerable group, who rely completely on food aid amid a striking food supply disruption. There is an urgent need for a sustainable solution to ensure refugees can afford the basic necessities needed for life and prevent the dangers to health food paucity threatens to these at-risk communities.

## Data Availability

Not applicable.

## Abbreviations

COVID-19: Coronavirus disease 2019
UNHCR: United Nations High Commissioner for Refugees
WFP: United Nations World Food Programme
UN: United Nations
DRC: Democratic Republic of Congo
SDG: Sustainable Development Goal
PTSD: Post-Traumatic Stress Disorder
